# Avulsion of the tuberosity of the calcaneus, an unusual injurie: case report

**DOI:** 10.11604/pamj.2016.23.132.9022

**Published:** 2016-03-25

**Authors:** Abdellatif Benabbouha, Abdelouab Jaafar

**Affiliations:** 1 Service de Chirurgie Orthopédique et Traumatologique I, Hôpital Militaire d'Instruction Mohamed V, Rabat, Maroc

**Keywords:** Avulsion, tuberosity, calcaneus

## Image in medicine

Avulsion calcaneal tuberosity fracture is an uncommon but potentially serious condition. Recent studies on the epidemiology of these specific fractures have demonstrated that avulsed calcaneal fractures account for 1.3% of all calcaneal fractures. It is therefore understandable that little has been written about these fractures. This type of injuries is usually caused by sudden muscular contraction of the Achilles tendon when the heel is flat on the ground. We report a very rare case of avulsion of the tuberosity of the calcaneus. A 47 year old male was admitted to the emergency department withpain and total functional impotence of his right lower limb after falling on the footduring a football game. The X-ray examination showeda displaced extra-articular fracture of the tuberosityof the calcaneus(A). The fixation of this fracture was carried out using an anterior-lateral approach, the large fragment wasstabilized with two kirschner wires (B). After reduction and healing, the patient recovered fully without clinical weakness of the triceps surae.

**Figure 1:**
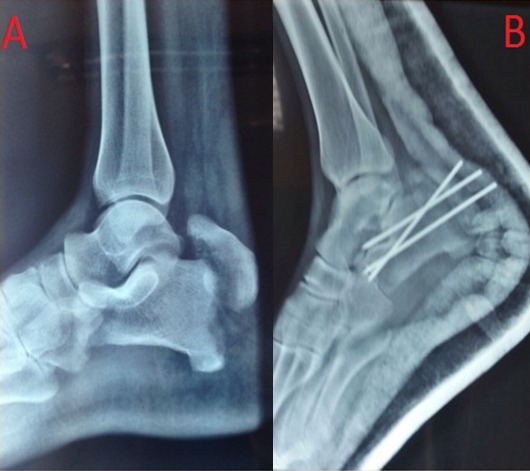
(A) radiographs of the right ankle demonstrating fracture of the tuberosity of the calcaneus; (B) X-ray on the first postoperative day

